# Evaluating the job shop scheduling problem on a D-wave quantum annealer

**DOI:** 10.1038/s41598-022-10169-0

**Published:** 2022-04-21

**Authors:** Costantino Carugno, Maurizio Ferrari Dacrema, Paolo Cremonesi

**Affiliations:** 1 Politecnico di Milano, Piazza Leonardo da Vinci, 32, Milan, Italy; 2 ContentWise, Via Schiaffino, 11, Milan, Italy

**Keywords:** Computational science, Computer science

## Abstract

Job Shop Scheduling is a combinatorial optimization problem of particular importance for production environments where the goal is to complete a production task in the shortest possible time given limitations in the resources available. Due to its computational complexity it quickly becomes intractable for problems of interesting size. The emerging technology of Quantum Annealing provides an alternative computational architecture that promises improved scalability and solution quality. However, several limitations as well as open research questions exist in this relatively new and rapidly developing technology. This paper studies the application of quantum annealing to solve the job shop scheduling problem, describing each step required from the problem formulation to the fine-tuning of the quantum annealer and compares the solution quality with various classical solvers. Particular attention is devoted to aspects that are often overlooked, such as the computational cost of representing the problem in the formulation required by the quantum annealer, the relative qubits requirements and how to mitigate chain breaks. Furthermore, the impact of advanced tools such as reverse annealing is presented and its effectiveness discussed. The results indicate several challenges emerging at various stages of the experimental pipeline which bring forward important research questions and directions of improvement.

## Introduction

Quantum computing is a computational architecture that uses quantum phenomena to offer a new paradigm that promises to revolutionize several scientific fields due to its superior computational power. Several algorithms have been developed for quantum computers that promise to pave the way for innovations in chemistry^1^, finance^2^, machine learning^3^ etc. The practical applicability of these algorithms is however limited by the constraints of the current gate-based quantum computers, which are noisy and have an insufficient number of qubits.

Another paradigm is *Quantum Annealing* (QA), which uses special purpose devices able to rapidly sample optimal solutions of optimization problems with a certain structure. As opposed to gate-based quantum computers, quantum annealers have a limited *application scope* but are both available with a much higher number of qubits and are more robust to noise. This has fuelled significant research from both industry and academia to explore the potential of this technology. Several formulations for important problems have been developed such as graph partitioning^4,5^, feature selection^6^, Support Vector Machines^7^ and Restricted Boltzmann Machines^8,9^. A number of important research questions remain open on how to successfully solve a problem on a quantum annealer, due to a multitude of factors: problem formulation, qubit connectivity, annealing schedule etc. These factors, however, are rarely discussed in papers that instead often focus on small proof-of-concept experiments.

This work studies a well-known problem in operation research: the Job Shop Scheduling Problem (JSSP). This problem has been selected for several reasons: (*i*) it is particularly difficult to treat for classical solvers^10^; (*ii*) it has a broad applicability and an important impact in cost mitigation for production chains; (*iii*) the formulation required to solve it on a quantum annealer is not trivial and has a computational cost that cannot be ignored; (*iv*) the problem requires a large number of qubits even for small instances.

The application of Quantum Annealing to the JSSP is not new, Venturelli et al.^11^ benchmarked the JSSP on a D-Wave Two quantum annealer, although the small number of qubits available—512 qubits—only allowed for a limited analysis. More recently, a heuristic procedure to split JSSP instances into smaller ones was proposed and tested on a D-Wave 2000Q quantum annealer^12^, which holds 2048 qubits. Lastly, several problems were successfully evaluated on a classical digital annealer that could handle up to 8192 variables, using the same JSSP formulation developed for quantum annealers, showing improved solution quality on some instances^13^.

This work aims to provide a balanced discussion of the potential as well as limitations of currently available Quantum Annealing technology applied to selected instances of the JSSP, and reports a step-by-step analysis of how to solve it on the new D-Wave Advantage quantum annealer, which possesses 5640 qubits. First, the problem formulation suitable for this device is presented and its computational cost discussed. Second, the impact of the *embedding* process of the problem into the quantum annealer is analyzed. Then, the paper reports an experimental analysis of the solution quality comparing both quantum and classical solvers on 116 problems of different sort. The paper also discusses the impact of several factors that can affect the solution quality, such as the number of solution sampled, the annealing duration, the chain strength, and the use of advanced controls such as Reverse Annealing. Overall, this work highlights both the prospects and the challenges of current available quantum technology applied to solving the JSSP, and puts forward several open research questions to highlight where further work is needed.

## Quantum annealing on a D-wave annealer

The term *Quantum Annealing*^14^ refers to a meta-heuristic that was proposed to minimize an objective function by leveraging quantum tunneling, i.e., the process that allows a particle to traverse a high energy barrier. Quantum Annealing shares a strong similarity with Simulated Annealing^15^, which instead is limited to the simulation of thermal fluctuations and, as such, is more susceptible to being trapped into local optima surrounded by high “thermal jumps”. In Quantum Annealing there is a certain probability to tunnel through the energy barrier escaping the local optima. Quantum Annealing leverages well known physics and has been successfully applied to several tasks by simulating the process on classical systems. The key idea of a quantum annealer is to build a special-purpose physical device that naturally exhibits quantum tunneling to find low-energy states.

Solving an optimization problem on a quantum annealer requires to represent its objective function in terms of the energy landscape of the quantum device, i.e., the *problem* Hamiltonian. The quantum annealer works by starting from an initial default configuration and then slowly evolving the physical system introducing the energy components of the desired problem. The total Hamiltonian of the system is represented as an Ising model, which allows to encode the energy as a function of the qubit states by setting the interactions among them. D-Wave quantum annealers use an Ising that represents interactions of at most two qubits:

$$\begin{aligned} {\mathscr {H}} = -\frac{A(s)}{2} \bigg (\sum _i \hat{\sigma _x}^{(i)}\bigg ) + \frac{B(s)}{2} \bigg (\sum \ h_i\hat{\sigma _z}^{(i)} + \sum _{i>j}J_{i,j}\hat{\sigma _z}^{(i)}\hat{\sigma _z}^{(j)}\bigg ) \end{aligned}$$

where $\sigma _x$ and $\sigma _z$ are matrices that represent the state of the system, i.e., the Pauli matrices, while *h* and *J* are parameters of the problem and allow to represent the desired energy function. The qubits are represented as spins, with eigenvalues $\{-1,+1\}$. The first term of the summation represents the initial state of the system, i.e., the *tunneling* Hamiltonian, where all qubits are in an equal superposition of both states. The second term is the final state of the system, the *problem* Hamiltonian, its lowest energy eigenvalue is the solution to the problem. The annealing process is controlled by functions *A*(*s*) and *B*(*s*), with $s \in [0,1]$ representing the stage of the annealing process, so that the problem Hamiltonian is introduced progressively. Note that on D-Wave quantum annealers the evolution schedule can be controlled by changing *s*. Under the ideal conditions of the *adiabatic theorem*^16^, if the annealing is slow enough the system remains in its lowest energy state throughout the process and reaches the ground state, i.e., the optimal solution, of the desired problem. Under a proper annealing schedule^17^, Quantum Annealing has been shown to be more effective than Simulated Annealing on problems such as Ising spin glasses^18^, the traveling salesman problem^19^, and some classes of non-convex problems^20^.

The Ising notation derives from statistical mechanics. An alternative formulation, closer to operations research, is called Quadratic Unconstrained Binary Optimization (QUBO), where the spins are replaced with binary variables $\{0,1\}$ via a simple substitution and the energy landscape is represented as a square matrix *Q*:

$$\begin{aligned} \mathscr {H_{\text{QUBO }}} = \sum _i Q_{i,i}x_i + \sum _{i<j}Q_{i,j}x_ix_j = x^{T}Qx \end{aligned}$$

The problem matrix Q can be visualized with the corresponding *problem graph*, where each variable represents a node associated to its linear term, the *bias*, and the quadratic terms between them are edges, associated to a weight called *coupling*.

**Figure 1:**
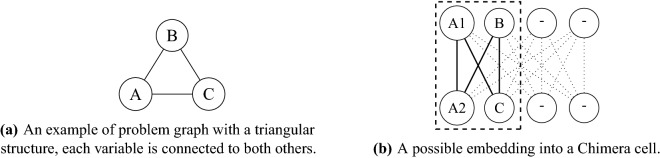
Example of how a problem graph, (**a**) is embedded in the Chimera annealer topology, (**b**) an earlier version of Pegasus. Each node represents a qubit and each edge a connection. Each qubit is connected to 4 others of the same cell and to others in different cells. The triangular problem graph cannot fit directly into the Chimera cell topology therefore the problem variable *A* becomes a logical variable represented by two different qubits *A1* and *A2* that will belong to a chain.
- *Sampling solutions* Given the embedding graph, the QPU can be programmed with the problem parameters (biases and couplers) and then the annealing process is performed. Although the adiabatic theorem requires a potentially very long annealing time to ensure the system remains in the ground state, this is not possible in practice as noise will push the state to higher energies. Therefore, a short annealing is performed which is repeated several times, in a stochastic process that samples from the energy distribution of the problem. In practice, finding the appropriate annealing time and number of samples depends on the problem itself and on the energy distribution, typical values are $10{-}100 \; \upmu \text{s}$ and $10{-}10^4$ samples.
- *Reverse annealing* While the traditional Quantum Annealing starts from an initial superposition and then evolves towards the desired problem, it is also possible to use more advanced strategies. In *Reverse Annealing* (RA) the process begins from an already available solution and the evolution is run backwards, reintroducing part of the tunneling Hamiltonian and therefore re-establishing a partial quantum superposition, *in order to further refine the initial solution*. In practice, the *s* term that controls functions *A*(*s*) and *B*(*s*) starts from 1, is reduced to a desired value, e.g., 0.45, and then is increased to 1 again. Although RA has been successfully applied in several tasks^23–29^, it is a substantially heuristic procedure, which depends on two critical parameters: the lowest value of the *s* parameter and the duration of the RA cycle. RA implements a local search^27^ in solution space that is governed by such parameters, which need to be chosen in a proper range in order to provide an improvement to the starting solution, i.e., high *s* values tend to conserve the initial solution, while lower *s* values explore a larger subspace but with a higher probability of obtaining a worse solution. The choice of optimal parameters was only recently explored with a machine learning approach using a Bayesian optimizer^28^, but in previous cases simpler approaches, such as trial-and-error^26^ and grid search^27^ have delivered practical improvements. Furthermore, RA refinements can be applied in succession, as a solution of a RA optimization can be used again in a new RA cycle, effectively implementing an Iterative Reverse Annealing (IRA) algorithm. In our work, in order to provide a simple and reproducible refinement component, we evaluate both a regular RA with different annealing time and also a simple two-step IRA with decreasing *s* terms.

The analysis reported in this paper are based on the following three steps:

- *Embedding a QUBO problem on a quantum annealer* In order to solve a problem on a quantum annealer, or Quantum Processing Unit (QPU) the problem graph must be mapped into the physical hardware, which has a limited number of qubits and connections between them. Therefore, it may be necessary to change the structure of the QUBO problem in such a way that it can be represented within the constrained topology of the quantum annealer. This process is called *minor embedding* and is computationally expensive, i.e., generally NP-hard, although polynomial-time heuristics exist. Improving the embedding algorithm is an important and active research topic^21,22^. A specific example is that the topology of the D-Wave 2000Q QPU, called *Chimera*, an earlier version of the more modern Advantage, does not have triangular connections (see Fig. 1). In this case, a logical variable is represented with multiple physical qubits, also known as a *chain*, that will split the needed physical connections. Clearly, for a solution to be consistent, all physical qubits in a chain must have the same value. To achieve that, new energy components are added to the problem ensuring a strong correlation between them. A chain is *broken* if at the end of the annealing process the qubits have different values. The strength of the correlation is a *parameter* that can be set according to a trade-off, if chosen too low many chains may break, if chosen too high it will hamper the ability of the qubits to change their state; both effects can lower the solution quality. The number of qubits needed for a given QUBO problem depends on several factors: the QPU topology, the QUBO connectivity and ultimately the embedding algorithm.

## Job shop scheduling

This section introduces the Job Shop Scheduling Problem (JSSP) and its QUBO formulation.

### Classical job shop scheduling

A job *shop* is an abstract model of a working location that is equipped with certain number of available *machines*. Each of these machines has a specific function in the productive chain of a *job*, which consists of an ordered series of *operations*. The machines can handle only one operation at a time and are able to work in parallel. The operations are executed on the machines according to a schedule. The aim of the optimization problem is to find the optimal schedule that minimizes the time required to complete all the jobs, i.e., the *makespan*. More formally, consider a problem instance that is made of a set of jobs $J = \{j_1, \ldots , j_n\}$ and a set of available machines $M = \{m_1, \ldots, m_k\}$. Each job consists of an ordered list of operations, $o_i$, each with its own time-to-completion, $p_i$: $j_1 = \{(o_1, p_1) \rightarrow (o_2, p_2) \rightarrow \ldots \rightarrow (o_s, p_s)\}$. Both operations and time-to-completion use a single global index. In a general setting, different jobs might have a different number of operations each with its own time-to-completion. In order to be executed, each operation needs to be assigned to one of the available machines, $\{o_1,\ldots,o_i\} \rightarrow \{(o_1,m_1),\ldots, (o_i,m_k)\}$. A solution of the problem is feasible when no operations are superimposed on the same machine and the order of the operations within each job is preserved. A solution is a schedule that associates the operations to their starting time and machine:

$$\begin{aligned} S: \{(o_1,m_1), \ldots, (o_i, m_k)\} \rightarrow \{(o_1,m_1,t_1), \ldots, (o_i,m_k,t_i)\}, \end{aligned}$$

where the index *i* represents the global operation index, *k* is the index of the machine and $t_i$ is the starting time of the operation as provided by the solver. The JSSP can be formulated as a general optimization problem: “find the schedule with the minimum total time to complete all operations”, i.e., the *makespan*, or as a decision problem: “is there a valid schedule with time less or equal to a certain given *timespan*?”.

This study focuses on the decision version of a selected cases of JSSP. First, jobs have fixed number of operations, equal to the number of jobs, i.e., $|j_n|=|J|$, where || represents the cardinality of a set. Furthermore, each operation can be executed only on a specific machine which is chosen when the problem instance is created (differently from the *flexible* JSSP^13^). This version of the JSSP therefore has as only degree of freedom choosing when each operation will start, taking into account the ordering of operations within a job and that there cannot be more operation running simultaneously on a machine.

### Quantum job shop scheduling

This study focuses on the decision version of the JSSP and uses the formulation introduced by Venturelli et al.^11^. The first step consists in formulating the decision problem as a Constrained Satisfaction Problem (CSP), asking whether there is a schedule that is shorter that a certain amount of time, i.e., the *timespan*. The QUBO model is composed of binary variables, each of which represents one operation per unit time, up to the *timespan*:

$$\begin{aligned} x_{it} = {\left\{ \begin{array}{ll} 1: \text {operation } o_i \text { starts at time } t_i \\ 0: \text {otherwise} \end{array}\right. } \end{aligned}$$

Due to this encoding, the total number of variables is ${\mathscr {N}}_{x} = {\mathscr {N}}_o \cdot timespan$, where ${\mathscr {N}}_o$ is the total number of operations which in this special case of JSSP corresponds to $|J|^2$. The CSP needs to satisfy three constraints. Since QUBO problems do not allow hard constraints, soft constraints are used by introducing a penalty for solutions that violate them and the QUBO is then constructed using penalty functions *h* associated to each of these constraints:

- *One start constraint*—An operation must start once and only once:
  $$\begin{aligned} \sum _t x_{it} = 1 \text { for each } i \rightarrow h_1(x) = \sum _i \bigg (\sum _t x_{it}-1\bigg )^2 \end{aligned}$$
- *Share machine constraint*—There can only be one job running on each machine at any given point in time:
  $$\begin{aligned} h_2(x)= & {} \sum _{(i,t,k,t')\in A_m \bigcup B_m}x_{it}x_{kt'} = 0 \text { for each } m \\ A_m= & {} \{(i,t,k,t'):(i,k) \in I_m \times I_m, i\ne k, 0\le t, t'\le T, 0 \le t'-t \le p_i\} \\ B_m= & {} \{(i,t,k,t'):(i,k) \in I_m \times I_m, i < k, t' = t, p_i>0, p_j > 0\} \end{aligned}$$
  where $p_i$ is the time-to-completion of operation $o_i$, $I_m$ is the set of all operations that are to be executed on machine, $A_m$ is used to enforce that the operations cannot start on the same machine when another one has already started on the same machine, and $B_m$ is used to ensure that two operations do not begin at the same time on the same machine.
- *Precedence constraint*—The constraint is based on the number of precedence violations between consecutive operations belonging to the same job. Given $s_{n-1}$ and $s_n$ the last operations of job $n-1$ and *n*:
  $$\begin{aligned} h_3(x) = \sum _{\begin{array}{c} s_{n-1}<i<s_n\\ t+p_i>t' \end{array}} x_{it}x_{i+1,t'} \text { for each } n \end{aligned}$$
  Finally, in order to penalize the solutions with larger *makespan*, a linear penalty $h_4(x)$ is added to the variable corresponding to the last starting time of the last operation of each job. This term is a diagonal and sparse contribution to the QUBO matrix. The resulting QUBO problem is then comprised of four terms, the constraints are each weighed with a penalty constant:
  $$\begin{aligned} {\mathscr {H}} = \alpha h_1(x) + \beta h_2(x) + \gamma h_3(x) + h_4(x) \end{aligned}$$

## Experimental methodology

The experimental analyses reported in this study rely upon the D-Wave cloud interface that provides access to different QPUs as well as various software tools and classical solvers. The results include two different QPUs: the D-Wave Advantage (5640 qubits with a mean 15 connections per qubit) and to a lesser extent the older D-Wave 2000Q (2048 qubits with a mean 6 connections per qubit). The minor embedding of the problem on the QPU is found with the *minor-miner* D-Wave library^30^. As classical solvers are reported a steepest descent greedy solver, Tabu Search and Simulated Annealing. It is also included a D-Wave proprietary hybrid quantum-classical solver that was developed for problems that do not fit on the QPU directly.

In order to represent the decision version of the JSSP with the QUBO formulation described in “Quantum job shop scheduling” section the JSSP is first represented as a binary Constraint Satisfaction Problem, which is then transformed into the QUBO form. This operation requires three steps. First, all the constraint inequalities are converted into equalities in a process called *binary expansion*, that might require the introduction of new auxiliary variables. Second, the final energy function is created combining all constraints as penalties. The weights of each constraint, i.e., $\alpha$, $\beta$ and $\gamma$, are selected according to a *penalty model*^16^ in order to ensure that unfeasible solutions, i.e., solution violating constraints, will always have a worse energy compared to optimal solutions that do not violate any constraint. In these experiments the penalties are chosen in such a way that each constraint violation increases the solution energy by 2. Finally, the QUBO problem is simplified by pruning variables associated to unfeasible solutions. Considering that within each job an operation cannot start until the previous ones are completed, for each operation *i* all variables $x_{it}$ with a *t* lower than the sum of the time-to-completion of all previous operations in the job are removed. The same is applied backwards by starting from the last operation of the job and assuming its starting time is the QUBO timespan.

In the experiments, QA is run for $20\; \upmu \text{s}$ with $10^3$ samples, except for two experiments in “Instances with an unknown optimal solution” section using $100\; \upmu \text{s}$ or $10^4$ samples. Reverse Annealing is benchmarked with $10^3$ samples from the best QA solution found, on two different time duration, $80\; \upmu \text{s}$ or $160\; \upmu \text{s}$, and two different annealing parameter threshold: the first iteration starts from the best QA solution and uses an intermediate tunneling field (i.e., *s* is brought to 0.55), then a second step with a higher field (i.e., $s=0.30$) is applied on the best RA solution.

## Results

Results are reported on two different categories of JSSP instances: (*i*) instances with a known optimal solutions; (*ii*) instances without a known optimal solution. The first group is designed to study the computational cost of the QUBO generation and the impact of the timespan, while the second to evaluate the solution quality in a more realistic setting and compare various solvers.

### Square instances with a known optimal solution

**Figure 2:**
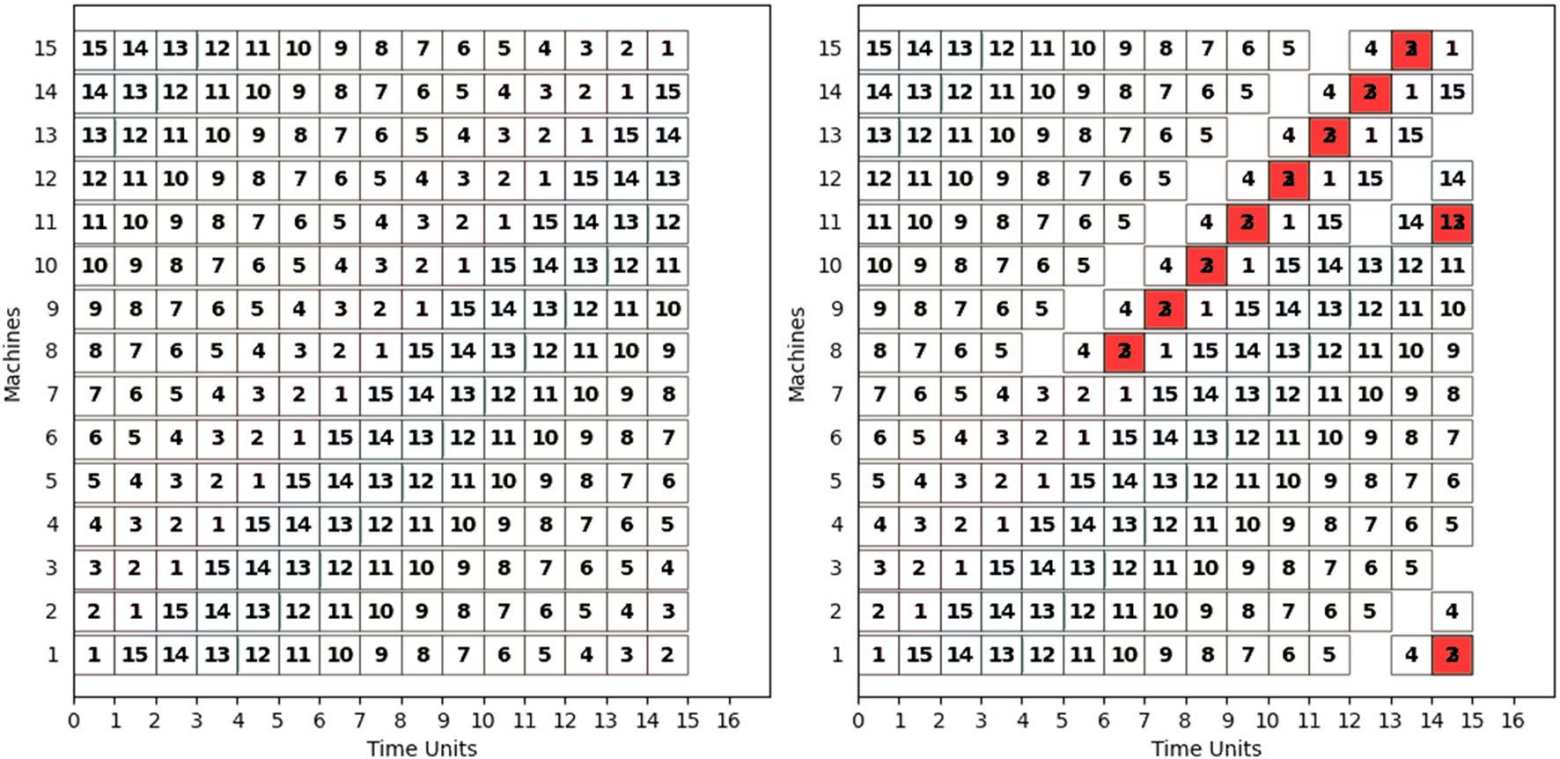
Solution schedules represented as a Gannt diagram for an instance of *size* 15 with a timespan set to 16. Each square represents an operation, the number inside indicates the job it belongs to. (*Left*) Quantum Annealing reaches the optimal solution. (*Right*) Simulated Annealing reaches an unfeasible solution. The operations that violate the share machine constraint by running simultaneously on the same machine are highlighted in red. Other operations violate the one start constraint and have not been scheduled.

The first set of JSSP instances is constituted by toy models aimed at evaluating the resource scaling of the quantum JSSP. The JSSP instances are designed to have a specific optimal solution, in which all machines are employed at the same time on an operation pertaining to consecutive jobs. In these JSSP instances the number of jobs |*J*|, of machines |*M*| and of operations per job |*O*| is such that $|J|=|M|=|O|=size$ with *size* a parameter used to define the instance. The instances are defined with a specific relation between the operations of the job and the machine they require. The first operation of each job requires a different machine, e.g., operation 1 of job 2 requires machine 2, operation 1 of job 5 requires machine 5. The pattern repeats in a cycle, i.e., operation *i* of job *n* is assigned to machine $(i+n-1)\ \mathbf {rem}\ |M|$, where |*M*| is the number of available machines and $\mathbf {rem}$ is the remainder operation. Furthermore, all operations are set with time-to-completion equal to 1 time unit, in order to minimize the number of variables needed to represent the time dimension. The solution of the problem instance can be visualized in Fig. 2 as a *Gannt* chart, a special bar chart that illustrates the schedule, as provided by a solver.

These instances are designed in such a way that the optimal makespan is known a-priori and is equal to the *size* parameter. As described in “Quantum job shop scheduling” section the JSSP formulation as decision problem requires to set a certain timespan. In the QUBO formulation, if the problem has a solution with makespan lower than the timespan set, then a solution with energy equal to zero is sure to exist. Note that if the timespan is equal to the optimal makespan the resulting QUBO matrix will simply be diagonal and the solution of the instance is trivial. This experiment studies how changing the timespan with respect to a fixed makespan affects the size of the instance and the solution quality. Problem instances are generated with *size* of 2 up to 26 and then their qubit requirements and solution quality is evaluated at increasing timespans, i.e., $size +1$ to $size + 6$.

#### QUBO generation

The first observation concerns the high computational cost of the QUBO generation. Even though the number of constraints grows quadratically with respect to the size parameter, the subsequent processing steps to combine the constraints and generate the penalty model result in an *exponential* time increase. This high computational cost is due to the penalty model which, given the CSP problem, combines all constraints and iteratively chooses the corresponding penalties, as described in “Experimental methodology” section. In particular, for instances larger than *size* 15 the most computationally intensive part is the selection of the penalty for the one start constraint, i.e., *h*1. Due to this, the maximum instance *size* that was generated is 26, which requires more than 8 h to be represented as QUBO. Lastly, although the combination of initial *size* and timespan would correspond to 150 QUBO instances, the analysis only includes the 85 that fit on the QPU. It should also be noted how the publicly available *penaltymodels* library used to generate the QUBO model tend to fail for more general problem instances, e.g., models that have operations with highly varying time-to-completion, models with a larger timespan etc.

#### Solution quality

**Figure 3:**
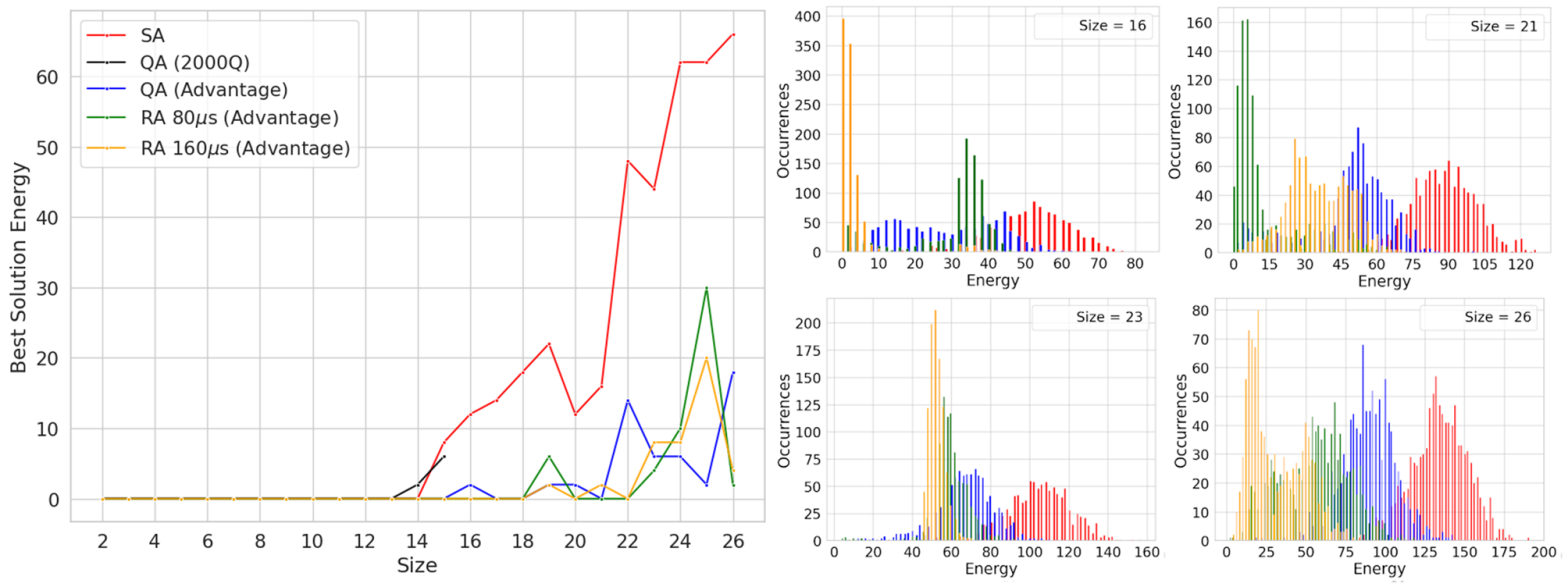
Solutions obtained with Simulated Annealing (SA), Quantum Annealing (QA) and Reverse Annealing (RA) on instances of increasing *size*, with timespan equal to $size+1$. Greedy and Tabu solutions were excluded from the plot due to their high energy. (*Left*) Comparison of the best solutions for each solver, i.e., those with the lowest energy. (*Right*) Distribution of all solutions sampled for selected cases, to compare their energy and the number of occurrences.

 The analysis focuses first on instances with $timespan = size + 1$ (see Fig. 3). Among the classical solvers Simulated Annealing is the best one, able to reach the optimal solution for instances up to *size* 14, while Tabu Search and greedy have poor solution quality. The greedy solver never reaches the optimal solution, for instances of *size* 2 the energy is 4, thus violating two constraints, while for *size* 26 the energy is 314. The Tabu Search performs slightly better and is able to reach the optimal solution up to *size* 10, but struggles on bigger instances, e.g., at *size* 26 the energy is 245.

Regarding the quantum solvers, QA on the Advantage QPU performs always on par or better than SA in all cases, reaching the optimal solution in instances where SA is not able to (i.e., $size = 15,17,18,21$). Concerning RA, increasing the RA duration generally improves the solution quality, which reaches optimality in several instances where QA alone is unable to (i.e., $size = 16, 20, 22$). As can be seen in the right part of Fig. 3, the samples obtained with QA and RA have a different distribution, in particular, QA produces samples that have longer tails while RA consistently produces a more peaked distribution that is shifted towards lower energies. Note that sometimes a longer RA duration produces a distribution peaking at higher energies.

The effects of the improvement in quantum annealer technology can be observed comparing the results of the more recent D-Wave Advantage with the previous generation 2000Q. The older QPU has lower qubit connectivity, which causes the formation of longer qubit chains requiring more qubits to represent the same instance and saturating the QPU much earlier, at instance *size* 15 (1173 qubits). It can be noted that the 2000Q solver is able to reach optimality up to instances with *size* 13. The new Advantage QPU can find the optimal solution of much bigger instances, up to *size* 21.

**Figure 4:**
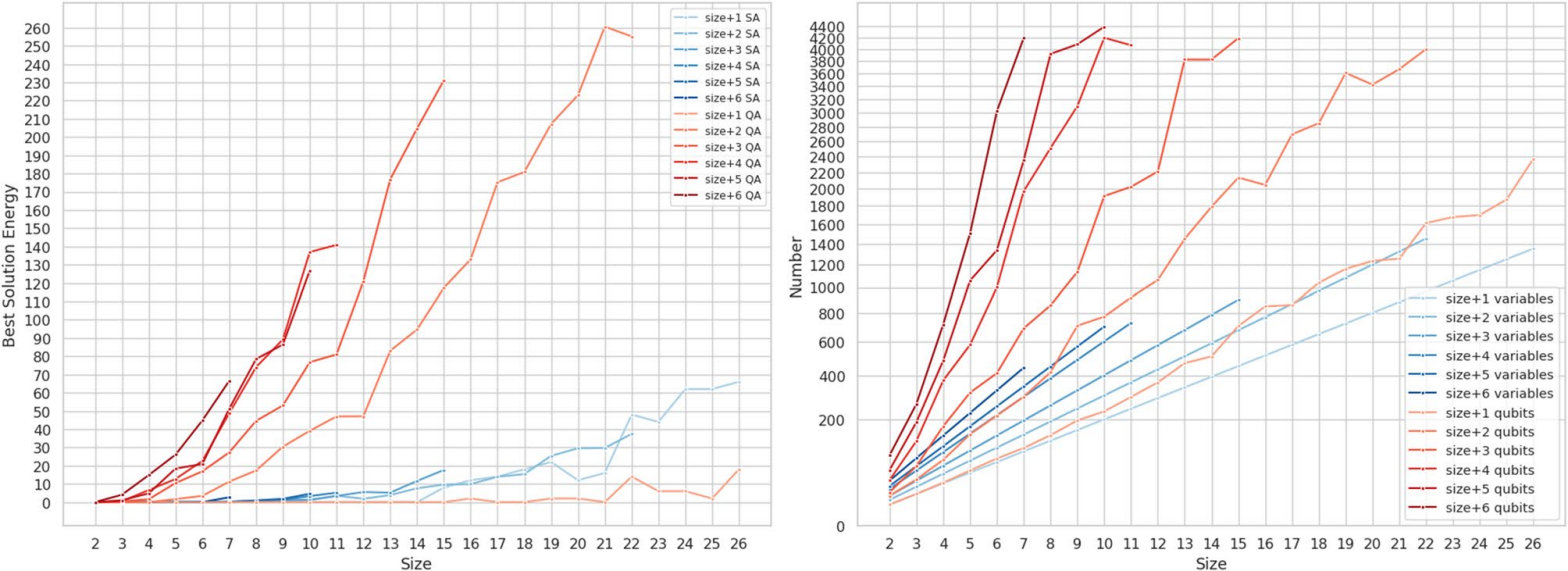
Visualization of the solution energy as well as the and number of variables and qubits required for JSSP instances with known solution of increasing *size* and timespan window, from $size+1$ to $size+6$. Results refer to the Advantage QPU, missing values indicate the instance did not fit on the QPU. (*Left*) Best solution obtained with Simulated Annealing (SA) and Quantum Annealing (QA). (*Right*) Semi-logarithmic plot of the QUBO variables and the qubits needed to embed them.

The left diagram in Fig. 4 compares the best solution energy of SA and QA as a function of the *timespan*, in the range $size+1 \ldots size + 6$. While for problem instances of timespan equal to $size +1$ QA prove to be a competitive strategy compared to SA, this rapidly changes as the timespan increases. The results indicate that Quantum Annealing is not able reach the quality of Simulated Annealing for instances with a large number of variables. **The right** diagram in Fig. 4 plots the number of variables and the number of qubits required to embed the QUBO problem as a function of the *timespan*. It can be seen that the number of qubits required to embed the instance increases substantially as the *timespan* increases and that there is a correlation between the increasing qubit requirements and the increasing energy of the best solution found. Also note that as the *timespan* becomes larger so does the gap between the number of qubits and the number of variables, up to a factor of 10. Note that for larger *timespan* values there is also a higher number of auxiliary variables, created to transform the inequality constraints into equality ones as described in “Experimental methodology” section. The number of auxiliary variables varies considerably: there are none in $size +1$ instances, while they reach $22\%$ of the overall variables for $size+6$ instances. These results indicate several important limitations and challenges: (*i*) the need to use a large number auxiliary variables to represent inequalities, (*ii*) high qubit requirements for larger instances, (*iii*) the need to choose a timespan that should be close to the optimal makespan, which would be very challenging to select in a real case. Results also indicate that RA is a promising strategy.

Due to the low solution quality reached in bigger instances, the rest of the analysis will focus on the application of advanced techniques such as Reverse Annealing and chain break mitigation in smaller instances that better fit the quantum annealer.

#### Chains and chain breaks

 As described in “Quantum annealing on a D-wave annealer” section, the minor embedding process to represent a QUBO problem on the QPU may create chains of physical qubits that represent the same logical variable. In a valid solution all physical qubits should have the same value but this may not happen in practice. If a chain is broken, the solution quality may quickly deteriorate. Regarding the instances with a known solution the chain break fraction is low for timespan equal to $size+1$ ($<1\%$) and progressively increases as *size* and timespan grow. For the largest embeddable instance of timespan equal to $size+6$ the chain break fraction has a mean of $37\%$, indicating that a substantial number of chains are broken. To address this, in order to improve the solution quality, a higher chain strength could be used as will be discussed in “Square instances with a known optimal solution” section.

### Instances with an unknown optimal solution

The instances with a known solution provide a simple set to benchmark the JSSP algorithm and investigate the solution space, but are unrelated to real-world problems. In order to analyze the JSSP performance on a quantum annealer in a more realistic scenario a second set of instances with an unknown optimal solution is introduced. The set is made of 31 square problem instances that are constituted of 4 jobs with 4 operations for each job, which are executed on 4 machines. As in the previous set of instances, each operation of a job requires a different machine, but in this case the assignment does not follow a precise cyclic pattern: the operations are assigned randomly among the machines, although enforcing that there will be one and one only operation per job scheduled on each machine, so they are still evenly distributed. Also, the time-to-completion of each operation is not fixed to 1 time unit anymore, rather it can be either 1 or 2 time units with equal probability.

Changing the operation time-to-completion and the assignment to a machine has an impact in both the QUBO generation and the solution space. Above all, the optimal makespan is unknown, and there is no direct way of choosing an appropriate timespan to be used in the formulation of the QUBO problem. As discussed in “Square instances with a known optimal solution” section, choosing a timespan higher than the optimal makespan causes the number of variables required to grow rapidly and the solution to degrade. On the other hand, a timespan set too low might make it impossible to generate the QUBO problem. In order to address this issue, in this experiment each JSSP instance is first solved with the open-source optimization library OR-tools^31^, which provides a valid solution to the JSSP minimizing its makespan, although it may not be the optimum one. This makespan is then used as the timespan to construct the decision version of the JSSP as QUBO problem as described in “Quantum job shop scheduling” section. Since all instances in this group are known to be solvable within the given timespan, by construction, the evaluation of the QUBO problem will be based on whether the solution found has energy zero.

#### QUBO generation

**Figure 5:**
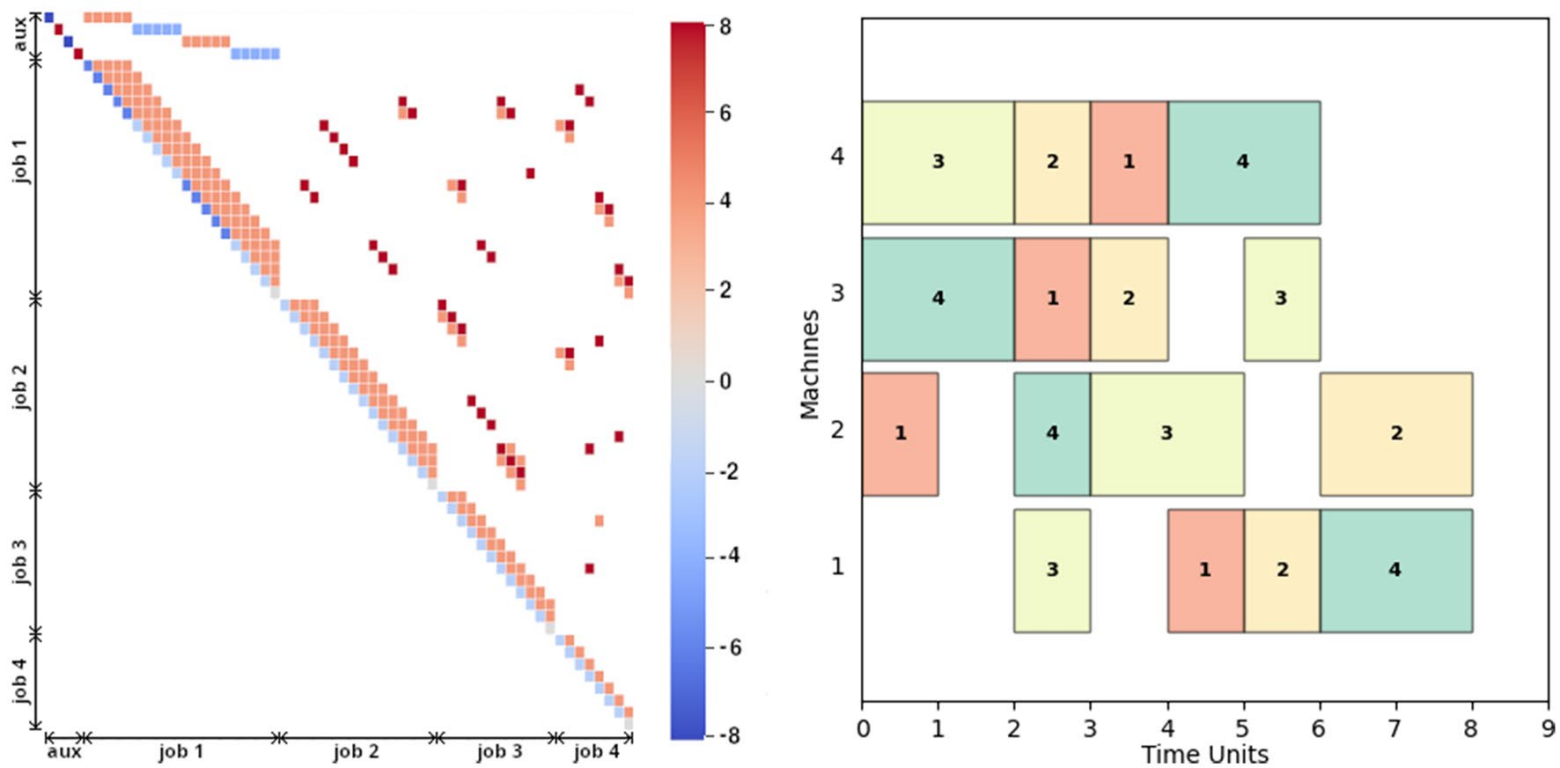
Visualization of a JSSP instance with unknown optimal solution. (*Left*), the QUBO matrix represented as heatmap, where each square represents a variable (operation per unit time). The color scale follows the bias and variance of each variable coupling. (*Right*) a Gannt diagram of a possible schedule obtained with OR-tools.

Due to the different setup, the generated set results in more heterogeneous problems, with a significant difference in variable number (i.e., 44–120) and qubit number (i.e., 65–505). Transforming inequalities into equalities requires a highly varying number of auxiliary variables: although none are present in 13 problem instances, the remaining ones require $4{-}20$ auxiliary variables, up to $16\%$ of the total variables. Figure 5 shows the QUBO formulation and the schedule of an instance of this problem set. The QUBO matrix, on the left, is represented as a heatmap which allows to visualize the effects of three different constraints in the QUBO generation: the one-start constraint causes the bias (the diagonal term, in blue) to be always of opposite sign compared to the coupling (off-diagonal terms, in red), the precedence constraint is enforced with a slight negative coupling among operations of the same job (in light red) and the share-machine constraint can be seen as the coupling of operations pertaining to different jobs, when present (in a darker red). The low number of the share-machine related couplings leads the matrix to be considerably sparse with a connectivity $\approx 12\%$. Moreover, the effects of variable pruning can be seen by comparing the QUBO heatmap with the sampled solution on the right. In particular, the first job needs the largest number of variables and further 4 auxiliary variables, connected only to other variables of the job itself. The last job needs much fewer variables since its operations last longer and their ordering allow less freedom in their allocation. The right part of Fig. 5 shows that by randomizing both the assignment of the operations to the machines and the operations time-to-completion the final schedule contains several gaps, where the machine is kept on hold. Due to this, the makespan depends on the arrangement of the operations in a non-predictable way.

The time required to generate the QUBO problems is generally in between 1 and 2 s for most instances under a 100 variables, although for the 4 largest problems the time requirement increases considerably to 20–260 s. This unexpected and abrupt increase is attributable to inefficient handling of the constraints in the penalty model library, which should be a focus of improvements for further works.

#### Solution quality

**Figure 6:**
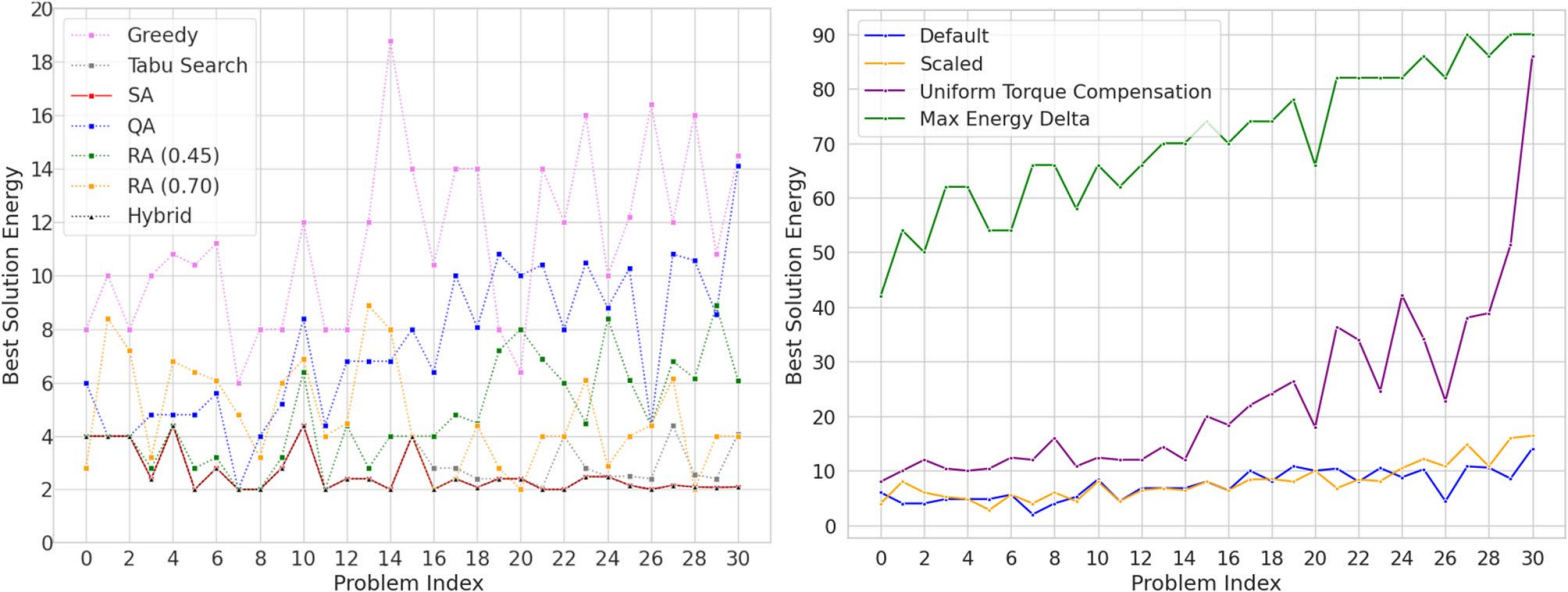
Energy plot of the best solution obtained with different methods: Simulated Annealing (SA), Quantum Annealing (QA) with a time durations of $20\; \upmu \text{s}$ and $10^3$ samples, Reverse Annealing at different tunneling field level (RA), classical (greedy and Tabu Search) and hybrid solvers. The instances on the x-axis are indexed by increasing number of variables. (*Left*) Solutions obtained for different problem instances. Note that Tabu Search, SA and Hybrid often overlap for small problem indexes. (*Right*) Solution obtained with QA by adopting different chain break mitigation strategies.

The left panel of Fig. 6 compares the solution quality of several solvers: two quantum solvers (Quantum Annealing and Reverse Annealing); three classical solvers (greedy strategy, Tabu Search and Simulated Annealing) and one hybrid quantum-classical solver. The greedy solver is the least effective, returning always the worst solution except for two instances. Tabu Search was effective when applied to small instances with a known solution described in “Square instances with a known optimal solution” section, and it also provides very good results in this new set of instances. Both Simulated Annealing and the hybrid solver produce results close to satisfy all the contraints (energy equal to zero) but are unable to full reach a satisfiable solution.

The quantum solvers generally provide a worse solution quality compared to the best classical ones. In particular, QA alone often struggles and is not able to reach the best solution. Increasing the number of annealing samples from $10^3$ to $10^4$ does not prove beneficial in this case and neither does increasing the annealing duration to $100\; \upmu \text{s}$. In this experiment, Reverse Annealing is employed with a time duration of $80\; \upmu \text{s}$ and the results indicate that it can provide a significant improvement to the solution quality compared to QA: for instances with a lower number of variables a limited transverse field is effective while a higher transverse field is preferable as the number of variables increases It is interesting to observe that increasing the QA duration to $100\; \upmu \text{s}$ does not provide improved solution quality, while the same amount of time but in a combination of $20\; \upmu \text{s}$ QA and $80\; \upmu \text{s}$ RA provides much better results. This indicates the importance of considering advanced tools like Reverse Annealing not simply as a possible fine-tuning step but rather as an integral part of the solution pipeline. Studying how to effectively leverage RA is an open and complex research direction.

#### Chains and chain breaks

As shown in “Square instances with a known optimal solution” section, the number of qubits necessary to embed the JSSP instances on a QPU differs considerably from the number of variables of the QUBO model. The embedding procedure maps each variable to one or more qubits, by forming chains. The length and the number of chains depend on the specific QPU topology, thus the same problem instance has different chain distributions for different QPUs.

**Figure 7:**
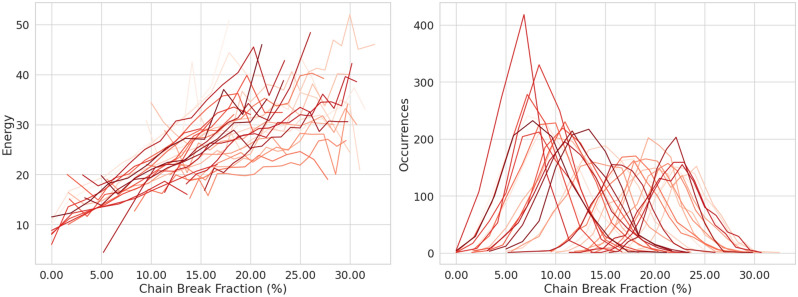
Chain break analysis of the 31 problems with unknown solutions, each problem is represented with a different red-yellow shade. (*Left*) Mean of the best solution energy of the $10^3$ samples of QA for each problem for a selected chain break fraction. (*Right*) Distribution of the chain break fraction in each problem, the occurrence counts the number of sampled solutions that shows a given fraction.

The right panel of Fig. 7 shows, for each instance, the distribution of the chain break fraction and we observe a typical Gaussian profile, with a considerable high mean (always greater than $5\%$ and averaging around $15\%$, among all instances) and a consistent standard deviation. The left panel shows the relationship between the **mean** chain break fraction and the mean energy of the solutions; there is a visible and almost linear correlation indicating that as the chain break fraction increases the solution quality worsens. The chain break fraction can be mitigated by adjusting the chain strength when the minor embedding is performed. The *default* *chain strength* is 2, but other heuristic strategies can be adopted. *Scaled*, in which the chain strength is scaled to the problem bias range, returns a *chain strength* $= 8$; *uniform torque compensation*, which attempts to compensate for random torque of neighboring qubits that would break the chain, results in a *chain strength* in the range of 15–22; *max energy delta*, which is based on the maximum increase in energy that is due to flipping a single variable and the obtained *chain strength* varies in the range 42–90.

The right panel of Fig. 6 shows the best solution found when applying each of the heuristic strategies to select the chain strength. As can be seen, the solution quality worsens when using uniform torque compensation and the max energy delta strategies. Both strategies are effectively able to reduce the number of chain breaks by a large margin, from 20–30% to 1–2%, despite this the best solution quality is reduced. This effect is due to the combination of two phenomena. First, qubits in strongly coupled chains tend to change their value less easily, making the system more rigid and less able to explore the solution space. Second, the energy component of the chain strength may become the dominant term in the problem overwhelming the other. Finding the appropriate chain strength is therefore a delicate task that requires a balanced trade-off.

## Conclusions

In this work we presented a step-by-step discussion on the practical implementation of the Job Shop Scheduling Problem and its evaluation using D-Wave quantum annealers, as a study on potential industry-relevant applications on currently available quantum devices. The benchmarks have been carried out on two different categories of problem instances, in order to highlight different challenges of a reliable and balanced evaluation. Firstly the apparently advantageous polynomial scaling in computational cost of the JSSP QUBO generation is hindered by the necessity of formulating a working cost function, i.e., choosing appropriate penalty terms for each constraint. A proper handling of the inequality constraints also produces additional auxiliary variables, which contribute to saturate the QPU resources, already limited by the process of qubit chaining. Secondly we note that in terms of solution quality, the quantum annealer is considerably effective compared to classical approaches on very constrained problem instances, while somewhat closely competitive on more realistic problem instances. Advanced techniques such as Reverse Annealing consistently alter the distribution of the solutions and prove particularly beneficial in more complex problems, although further research would be needed to assess how to leverage more effectively advanced controls of the quantum annealer. We acknowledge that the burden of the generation of a medium sized QUBO can be partially mitigated by using a specialized middleware^32,33^ or an improved penalty model. Relatively small instances were analyzed in this study, which already present several challenges, alongside encouraging prospects. A promising technique that could allow to evalute larger QUBOs proficiently, consists in splitting a large instance into several smaller sub-QUBOs and solve these smaller pieces separately, merging together the different results to obtain the full instance solution^34^. Although such evaluations would carry additional research questions, we hope that this work could be a good starting point for further benchmarks towards a real-world advantage in operations research applications.

## Data Availability

The code necessary to replicate this work is available on GitHub, at https://github.com/qcpolimi/JobShopScheduling. The public repository also includes further visualizations from this study.
